# Five Things to Know about Genetically Modified (GM) Insects for Vector Control

**DOI:** 10.1371/journal.ppat.1003909

**Published:** 2014-03-06

**Authors:** Luke Alphey, Nina Alphey

**Affiliations:** 1 Oxitec Limited, Milton Park, Oxford, United Kingdom; 2 Department of Zoology, University of Oxford, Oxford, United Kingdom; 3 Mathematical Ecology Research Group, Department of Zoology, University of Oxford, Oxford, United Kingdom; University of Wisconsin Medical School, United States of America

## Five Things to Know about Genetically Modified (GM) Insects for Vector Control

### 1. Why (and how to) use GM vectors for vector control?

Vector-borne diseases cause immense suffering and economic damage. Vector control remains a key element of mitigation and control strategies, particularly for pathogens such as dengue viruses for which there are no specific drugs or vaccines. Yet existing vector control tools are limited; toxic chemicals are the mainstay but difficult to deliver due to vector behaviour, emerging resistance, and/or environmental concerns. Genetically modified vectors—presently only mosquitoes—offer complementary new approaches to integrate with the best existing methods. Modified mosquitoes will actively seek out wild mosquitoes as mates, with high species specificity and minimal off-target effects.

Within this overall scheme, many different genetic modifications have been proposed, all delivered via this mating-based mechanism (“vertical transmission”). These may be classified according to the persistence of the modification: “self-sustaining” genetic systems are intended to persist or spread invasively in the wild population after an initial release period, while “self-limiting” systems will disappear relatively rapidly unless maintained by more releases. Another classification is by intended effect: “population suppression” strategies aim, like most current vector control programmes, to reduce the number of vector mosquitoes in the target area, while “population replacement” strategies aim to reduce the ability of affected mosquitoes to transmit specified pathogens, with any reduction in total number of mosquitoes being incidental. In either case, the intended result is fewer competent vectors, thereby reducing the force of infection. In computer simulations, several such strategies are capable of eliminating transmission in the programme area.

These approaches are not entirely new. Some proposals [1] are simply applications of modern genetics to improve on the classical Sterile Insect Technique (SIT) [2], in which radiation-sterilised insects are released to mate with wild counterparts and thereby reduce the reproductive potential of the target pest population, leading to suppression or even local elimination. SIT has been used successfully on large and small scales against some major agricultural pests. This close relationship to an existing method means that the rollout, use, strengths, and weaknesses of such self-limiting population suppression strategies are fairly predictable and well understood. For self-sustaining strategies, looser analogies may be drawn with classical biological control, in which an exotic predator or parasite is introduced with the intention that it should establish permanently and thereby help control the pest. This analogy highlights both key strengths of self-sustaining systems—potential long-term benefit without further human action—and weaknesses—relative lack of control post-release—relative to self-limiting ones. Simulation modelling is a vital tool to inform strain development and risk assessment and mitigation, especially of the more invasive self-sustaining systems in which release is essentially irreversible.

### 2. How GM mosquitoes are made

**Figure 1:**
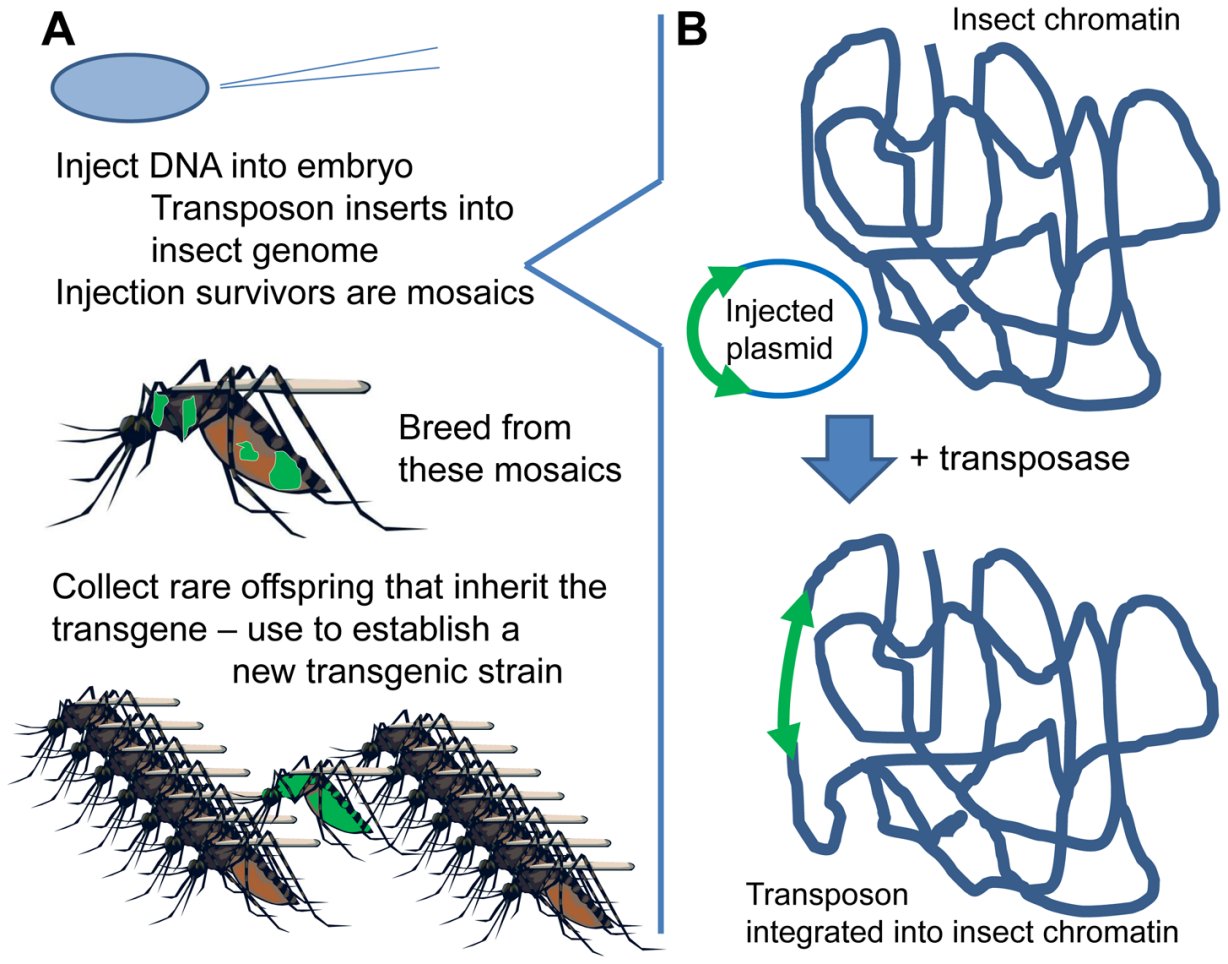
Creating a new transgenic strain. (A) DNA is injected into insect eggs; offspring of injection survivors are screened for presence of the marker gene indicating transgene presence. (B) Transposase-mediated transgene insertion.

Inserting DNA into an insect's chromosome (“genetic transformation”) is currently accomplished by means of a transposon-based system (Figure 1) [3]. The DNA of interest is placed between the ends of a suitable transposon (e.g., *piggyBac*, *Minos, mariner*, or *Hermes*). This plasmid is micro-injected into embryos, along with “helper” transposase (as mRNA or plasmid). The helper transposase acts on the transposon ends and, at very low but nonzero frequency, causes the transposon to “jump” from the injected plasmid into the insect's chromosomes (Figure 1B). Each transposon has its characteristic insertion site (e.g., TTAA for *piggyBac*), but these are present in so many copies in the genome that insertion is essentially random. The inserted DNA, lacking its own transposase gene (“non-autonomous” transposon), is then stably integrated in the insect's genome.

The DNA is injected into syncytial embryos, an early developmental stage before cells form, in which there are many nuclei within a shared cytoplasm. The injected DNA can therefore reach any of the nuclei. If some germline cells are transformed, then the offspring arising from them will carry the inserted DNA in all their cells—a new, transformed individual from which a transformed line can be established by simply breeding (Figure 1A). A marker gene, usually a fluorescent protein, is incorporated into the genetic construct to identify these rare, transformed insects. The transformation process is now routine in several important vector mosquitoes, such as *Aedes aegypti*, *Anopheles stephensi*, and *An. gambiae*.

Transformation by homologous integration is not available for mosquitoes, though long–recognition-site nucleases may facilitate this. However, site-specific integration has been developed by inserting “docking sites” on transposons and then allowing targeted integration into these engineered sites [4]. This is very valuable for some purposes: for example, comparing the effects of two different constructs. Such experiments were previously confounded by “position effect:” regulatory elements in the flanking chromatin interact with inserted DNA and affect its expression, so the same construct in different locations often gives slightly different expression and phenotype. This can be advantageous by allowing the experimenter to fine-tune expression simply by screening a panel of insertion lines, but it causes problems for other types of experiments.

Other approaches to genetic modification [5]–[9], for example, artificial infection with maternally transmitted *Wolbachia pipientis* bacteria or paratransgenesis (genetically engineering the vector's symbionts), are beyond the scope of this article.

### 3. Progress in the field

Some proposed strategies exist only as attractive-looking simulations [10], others as proof-of-principle in *Drosophila* or mosquitoes, but some have already entered advanced cage and field trials. This is remarkable progress given the low investment in this area by funding agencies, relative to insecticides, drugs, or vaccines, and how recently the molecular genetic tools and techniques were invented.

The first field trials of GM mosquitoes involved a self-limiting, population-suppression, sterile-male system known as RIDL (Release of Insects carrying a Dominant Lethal genetic system) [11]. Trials have shown that lab-reared, genetically engineered *A. aegypti* RIDL males can compete successfully for mates in the field [12], have similar field performance (e.g., longevity) to an unmodified comparator strain [13], and that sustained release of such males can suppress a target field population [14]. These data are extremely encouraging for the further development of this RIDL approach and, also, for GM mosquito methods generally.

SIT-related methods require the release of considerable numbers of modified male mosquitoes. Though the economics and timescales needed to achieve significant disease reduction look highly attractive [15], [16], in some instances even more powerful methods may be desirable. More invasive genetic systems are being developed, which models predict would require far fewer mosquitoes to be released [17]–[19]. Although the costs of post-release monitoring and stewardship of self-sustaining systems should not be underestimated, these aggressive systems are likely to be far cheaper to deploy against very widely distributed pests and species complexes, the main trade-off being lack of control post-release. Cost of development is also higher, but for all these systems development is a one-time cost that looks trivial relative to the potential benefit.

### 4. It's not just the genetics

Developing promising genetic strategies and modified strains that embody them is a necessary step, but far from sufficient. New technologies need to win public acceptance. The idea of dealing with a dangerous mosquito by releasing more of them is hardly intuitive! This is compounded by public concerns over the use of genetic approaches. Regulatory systems are also challenged by these new methods; for both the public and regulators, “self-sustaining” methods intending to lead to the permanent presence of novel genetic traits in wild vector populations may be especially problematic. Recombinant DNA methods may have an advantage in that frameworks already exist in many countries to regulate environmental use of genetically engineered organisms, albeit typically written with GM crops in mind. Mechanisms for appropriate oversight of other (non-recombinant) methods of genetic modification may be harder to devise.

Even from a purely technical perspective, success depends on more than good genetics. Efficient methods for rearing high-quality mosquitoes are required, particularly for those methods requiring relatively large numbers to be released. A key limitation for developing such methods is the lack of good proxy measure for field quality. Improved understanding of vector ecology would also allow more efficient use of these genetic tools. Ultimately, “success” will be defined in terms of epidemiological outcomes, and a further challenge is how best to demonstrate the efficacy of genetic vector control methods in reducing disease transmission [20].

### 5. Applications and limitations—What makes a good target?

Genetic methods depend on the vertical (parent-to-offspring) inheritance of one or more novel traits. Mating between modified vectors and wild conspecifics is therefore crucial to all such methods. However, mating barriers may exist between populations, or even different cryptic species with complete barriers to gene flow. In addition to natural mating barriers, selection and genetic drift may cause artificially reared laboratory strains to diverge significantly relative to wild strains, leading to poor mating. Highly invasive genetic systems may be able to cross incomplete hybridisation barriers; this spreading ability may be a significant advantage in such settings, while simultaneously a source of concern from a regulatory perspective.

More prosaically, the manipulations associated with introducing the modified trait likely require that the target species be reasonably easily reared in the laboratory. Though not a fundamental limitation, the need to develop adequate rearing methods would add to the time and cost of developing genetic control tools in species where such methods are not already established.

Genetic control methods target a single species (or species complex). From an economic perspective, this may be highly attractive when a single—or perhaps two or three—dominant vector species can be targeted, less so, elsewhere. Dengue, for which *A. aegypti* is the dominant vector worldwide, clearly fits this criterion. If multiple vector species are present but some are well controlled by other methods, genetic control may be useful as part of integrated vector management; this may apply in some African malaria contexts. Though development has so far focused on vectors of human diseases, many vectors of livestock and plant diseases will likely also prove amenable to genetic control methods.

More generally, genetic methods should not be seen as “magic bullets” that will single-handedly solve a problem but rather as new and powerful approaches with specific strengths and limitations. That such new approaches are needed seems beyond doubt. These should be combined with existing methods to provide programme managers with more options for effective, safe, and sustainable vector control. The prospects for genetic methods to contribute to effective control of vector-borne diseases look very promising.
